# Molecular mechanism and therapeutic implications of selinexor (KPT-330) in liposarcoma

**DOI:** 10.18632/oncotarget.13485

**Published:** 2016-11-21

**Authors:** Manoj Garg, Deepika Kanojia, Anand Mayakonda, Jonathan W Said, Ngan B Doan, Wenwen Chien, Trivadi S Ganesan, Linda Shyue Chuang Huey, Nachiyappan Venkatachalam, Erkan Baloglu, Sharon Shacham, Michael Kauffman, H. Phillip Koeffler

**Affiliations:** ^1^ Cancer Science Institute (CSI) of Singapore, National University of Singapore, Singapore; ^2^ Department of Medical Oncology and Clinical Research, Cancer Institute (WIA), Adyar Chennai, India; ^3^ Department of Pathology and Laboratory Medicine, David Geffen School of Medicine, Los Angeles, CA, USA; ^4^ Karyopharm Therapeutics Inc, Newton, MA, USA; ^5^ Division of Hematology/Oncology, Cedars-Sinai Medical Center, University of California Los Angeles, School of Medicine, Los Angeles, CA, USA; ^6^ National University Cancer Institute, National University Hospital, Singapore, Singapore

**Keywords:** selinexor, IGFBP5, xenograft, cell cycle

## Abstract

Exportin-1 mediates nuclear export of multiple tumor suppressor and growth regulatory proteins. Aberrant expression of exportin-1 is noted in human malignancies, resulting in cytoplasmic mislocalization of its target proteins. We investigated the efficacy of selinexor against liposarcoma cells both *in vitro* and *in vivo*. Exportin-1 was highly expressed in liposarcoma samples and cell lines as determined by immunohistochemistry, western blot, and immunofluorescence assay. Knockdown of endogenous exportin-1 inhibited proliferation of liposarcoma cells. Selinexor also significantly decreased cell proliferation as well as induced cell cycle arrest and apoptosis of liposarcoma cells. The drug also significantly decreased tumor volumes and weights of liposarcoma xenografts. Importantly, selinexor inhibited insulin-like growth factor 1 (IGF1) activation of IGF-1R/AKT pathway through upregulation of insulin-like growth factor binding protein 5 (IGFBP5). Further, overexpression and knockdown experiments showed that *IGFBP5* acts as a tumor suppressor and its expression was restored upon selinexor treatment of liposarcoma cells. Selinexor decreased aurora kinase A and B levels in these cells and inhibitors of these kinases suppressed the growth of the liposarcoma cells. Overall, our study showed that selinexor treatment restored tumor suppressive function of *IGFBP5* and inhibited aurora kinase A and B in liposarcoma cells supporting the usefulness of selinexor as a potential therapeutic strategy for the treatment of this cancer.

## INTRODUCTION

Liposarcoma is the most common type of soft-tissue tumor, accounting for 24% of extremity and 45% retroperitoneal soft-tissue sarcomas [1–3]. According to the World Health Organisation and others, liposarcoma is currently sub-classified into five subtypes including (1) well-differentiated; (2) dedifferentiated; (3) myxoid; (4) pleomorphic; and (5) round/mixed [1–3]. Complete surgical resection is the main regime for treatment of localized liposarcoma, but metastatic disease is incurable by surgery resulting in a poor overall survival [1–5]. Radiation and chemotherapy have limited value in treatment of metastatic liposarcoma [6, 7]. Therefore, a compelling need exists for new therapeutic targets to improve clinical care of these patients.

Transport of specific proteins between the nucleus and cytoplasm is a fundamental process for maintaining cell proliferation and apoptosis of normal and tumor tissues. Exportin-1 (*XPO1*) is a well-known nuclear export receptor responsible for transporting more than 220 cargo proteins as well as several RNA molecules from the nucleus to the cytoplasm [8–10]. *XPO1* recognizes cargo proteins through a hydrophobic, leucine-rich nuclear export signal, which is dependent on the RanGTP/GDP axis [8–10]. *XPO1* is the sole nuclear exporter of many tumor suppressive and growth-stimulatory proteins including *p21*, *p27*, *p53*, p73, *STAT3*, *BRCA1*, *FOXO*, *CDKN1A*, *RB1*, *IkB*, *APC*, *NPM1* and Survivin [11–14]. *XPO1* is up-regulated in different human malignancies such as leukemia [15], lung cancer [16], hepatocellular carcinoma [17], melanoma [18], as well as multiple myeloma [19]; and its overexpression is correlated with poor prognosis, resistance to chemotherapy and short survival [12], [15], [19]. Leptomycin B was the first well known natural *XPO1* inhibitor that suppressed the growth of several human cancer cell lines [20]. However, this drug had significant toxicity and a narrow therapeutic window in preclinical animal models, as well as in phase 1 human clinical trial [21]. Recently, novel orally bioavailable small molecules known as Selective Inhibitors of Nuclear Export have been developed. These inhibitors specifically and reversibly bind to residue Cys528 in the cargo-binding groove of *XPO1*. Selinexor (KPT-330) is the most advanced oral inhibitor of *XPO1*. Phase I/II human clinical trials have indicated that selinexor is well-tolerated and has a favorable outcome in patients with either relapsed or refractory acute myeloid leukemia (NCT01607892) and solid tumors (NCT01607905, NCT01896505) [22] (www.clinicaltrials.gov). In this current study, therapeutic potential of selinexor was examined against liposarcoma both in cell culture and in a murine xenograft model. Selinexor significantly inhibited cellular proliferation and induced cell cycle arrest and apoptosis of liposarcoma both *in vitro* and *in vivo*.

## RESULTS

### *XPO1* expression in liposarcoma samples and cell lines and *XPO1* silencing in liposarcoma cells

**Figure 1:**
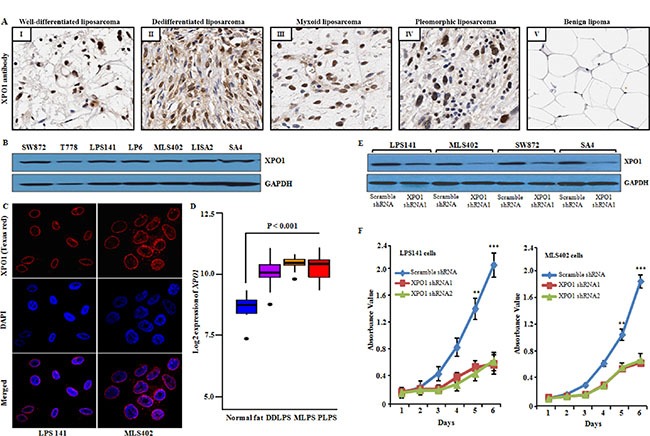
Expression of XPO1 in human liposarcoma tissue and cell lines, and XPO1 knockdown in liposarcoma cells (**A**) XPO1 protein expression was examined in liposarcoma tissue and benign lipoma using immunohistochemical analysis. Representative photomicrographs showed nuclear expression of endogenous XPO1 in well-differentiated liposarcoma (I), dedifferentiated liposarcoma (II), myxoid liposarcoma (III) and pleomorphic liposarcoma (IV) tissue samples, whereas benign lipoma (V) showed either very less or no reactivity (original magnification, X200; objective, X20). (**B**) Western blot analysis of liposarcoma cell lines probed with a XPO1 antibody (band 123 kDa, corresponding to the size of XPO1 protein). GAPDH used as the loading control. (**C**) Nuclear localization of XPO1 protein (red color) in fixed/permeabilized liposarcoma cell lines. DAPI (blue color) was used to stain nuclei. (**D**) Microarray data (GSE21122) from GEO database for samples of 46 dedifferentiated liposarcoma (DDLPS), 20 myxoid liposarcoma (MLPS), 23 pleomorphic liposarcoma (PLPL) and 9 normal fat tissue; approximately 90% of samples showed significant (*P <* 0.001) upregulation of XPO1 compared to normal fat samples. (**E**) Western blot confirmed knockdown of XPO1 protein in LPS141, MLS402, SW872 and SA4 cells infected with *XPO1* shRNA1 compared to scrambled shRNA. GAPDH antibody was used to assure equal loading of lysates. (**F**) *XPO1*knockdown suppressed cell growth of LPS141, MLS402. Data represent mean ± SD; *n* = 4. **P ≤ 0.001, ***P ≤ 0.0001.

To determine the expression of endogenous XPO1 protein in liposarcoma patient samples, we first performed XPO1 staining on 20 well-differentiated liposarcoma, 13 dedifferentiated liposarcoma, 13 myxoid liposarcoma, 2 pleomorphic liposarcoma and benign lipoma tissue sections (Figure 1A) and analyzed the staining levels by H-score method. A total of 58% of liposarcoma samples showed strong nuclear staining (H-score value > 199), 29% had moderate nuclear staining (H-score value > 99), and 13% had weak nuclear staining (H-score value 0 – 99) (Supplementary Figure S1A). In contrast, very weak or negative immunoreactivity of XPO1 was observed in benign lipoma tissues (Figure 1A). Western blot analysis showed XPO1 protein expression in liposarcoma cell lines of different histological subtypes (undifferentiated, SW872; well differentiated, T778; dedifferentiated, LPS141, LP6; myxoid, MLS402; poorly differentiated, LISA-2; SA4) (Figure 1B). Furthermore, immunofluorescence analysis revealed strong nuclear membrane localization of XPO1 protein in fixed, permeabilized LPS141, MLS402, SW872 and SA4 cells (Figure 1C and Supplementary Figure S1B). In addition, *XPO1* expression was examined in different subtypes of liposarcoma, using microarray database GSE21122 comprising 46 dedifferentiated liposarcoma, 23 pleomorphic liposarcoma, 20 myxoid liposarcoma samples and 9 normal fat samples. We observed that 90% of liposarcoma samples showed significantly (*P* < 0.01) higher expression of *XPO1* compared to normal fat (Figure 1D). These results demonstrated that XPO1 is prominently expressed in different histological subtypes of liposarcoma. To examine the biological role of *XPO1* in liposarcoma, the gene was first suppressed using shRNA targeting to *XPO1* resulted in 70–90% silencing of *XPO1* protein in liposarcoma cells (LPS141, SW872, MLS402 and SA4) compared to scramble shRNA as shown by western blot analysis (Figure 1E). This led to significant inhibition of cellular proliferation of these liposarcoma cells compared to scramble shRNA (Figure 1F, Supplementary Figure S1C).

### Inhibition of *XPO1* decreased cellular growth of human liposarcoma cells

**Figure 2:**
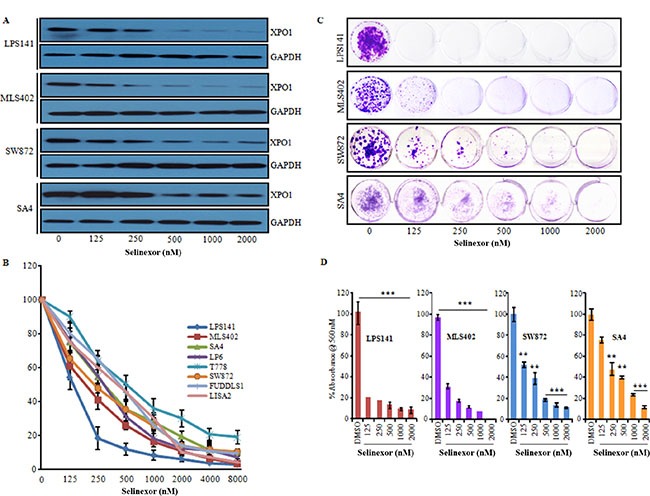
Selinexor significantly suppressed growth of liposarcoma cell lines in liquid culture (**A**) LPS141, MLS402, SW872 and SA4 cells were treated with either diluent (DMSO) or increasing concentrations of selinexor (0, 125, 250, 500, 1000 and 2000 nM, 24 h). Cell lysates were analyzed by western blots for XPO1 protein (GAPDH, internal control). (**B**) Selinexor inhibited cell proliferation of liposarcoma cell lines in a dose-dependent manner. Cells were cultured in the presence of selinexor at the indicated doses for 72 hours, and cell growth was assessed by MTT assay. Data represent mean ± SD; *n* = 4. (**C** and **D**) Selinexor suppressed clonogenic growth. Cells were treated with indicated concentration of selinexor for 24 h, washed and then allowed to form colonies for 14 days. Colonies were stained with crystal violet and dissolved in DMSO. Representative photomicrograph (C) and quantitative analysis showed a reduction in clonogenic growth (D). Data are expressed as mean values ± SD of at least four independent experiments. **P ≤ 0.001, ***P ≤ 0.0001.

Next, efficacy of selinexor to inhibit *XPO1* expression of LPS141, SW872, MLS402 and SA4 cells was examined after treating with increasing concentrations of selinexor (0–2000 nM, 24 h). Selinexor inhibited XPO1 protein levels in a dose-dependent fashion in all four liposarcoma cell lines at 24 h (Figure 2A). However, selinexor treatment did not decrease *XPO1* mRNA levels (data not shown) suggesting that the drug effected protein levels of XPO1. Further, a panel of liposarcoma cell lines representing different histological subtypes were treated with selinexor also caused a dose-dependent decrease in cell viability (IC50, ranged between 100–500 nM) (Figure 2B) and also markedly inhibited the clonogenic capacity of liposarcoma cells in a dose-dependent manner (Figure 2C and 2D).

### Selinexor induced apoptosis and cell cycle arrest in liposarcoma cells

**Figure 3:**
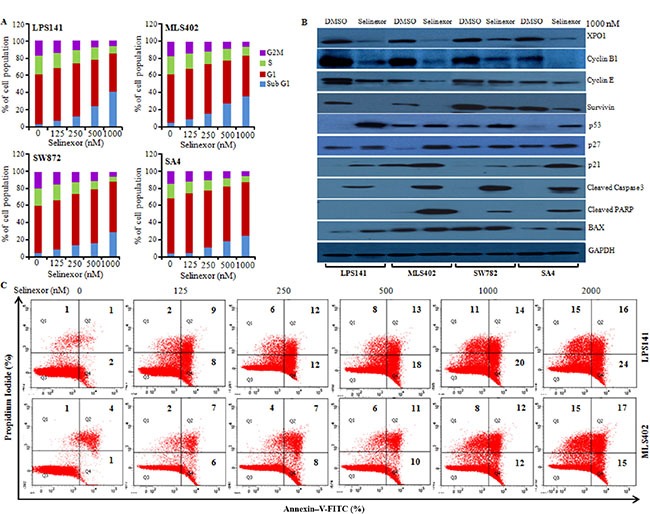
Selinexor induced cell cycle arrest and apoptosis in liposarcoma cell lines (**A**) Liposarcoma cells were incubated with either diluent control (DMSO) or different concentrations of selinexor for 48 h, stained with propidium iodide (PI) to determine cell cycle profiles using flow cytometric analysis. Data displayed as histogram (mean of three independent experiments). (**B**) Liposarcoma cells were cultured in presence of selinexor (1000 nM) for 24 h. Cell lysates were prepared and subjected to western blot analysis using different antibodies (GAPDH, loading control). (**C**) Apoptosis of liposarcoma cells after 48 h exposure to different concentration of selinexor. Cells were stained with Annexin V-FITC and PI and analyzed by flow cytometry. Percentage of apoptotic cells either Annexin V + PI or both is displayed in each treatment group of three independent experiments.

Liposarcoma cell lines were treated with increasing concentrations of selinexor (0–1000 nM) or diluent control for 24 h, and cell cycle distributions were determined by staining with propidium iodide (PI). Selinexor significantly lead to accumulation of cells in the G1 phase; and reduced cells in the S and G2M phase (Figure 3A). Concomitantly, selinexor (1000 nM, 24 h) prominently decreased protein levels of cyclin B1, cyclin E, survivin and increased levels of p21, p27 and p53 (Figure 3B). Flow cytometric analysis demonstrated a dose-dependent increase in the percentage of apoptotic cells (PI^high^/AV^high^), and a parallel decrease in viable liposarcoma cells (PI^low^/AV^low^) after culture with selinexor (0–2000 nM, 24 h) (Figure 3C). Furthermore, western blot analysis showed that selinexor (1000 nM, 24 h) increased cleaved caspase-3 and cleaved PARP as well as increased BAX protein expression compared to vehicle control (Figure 3B).

### Selinexor inhibited growth of liposarcoma xenografts

**Figure 4:**
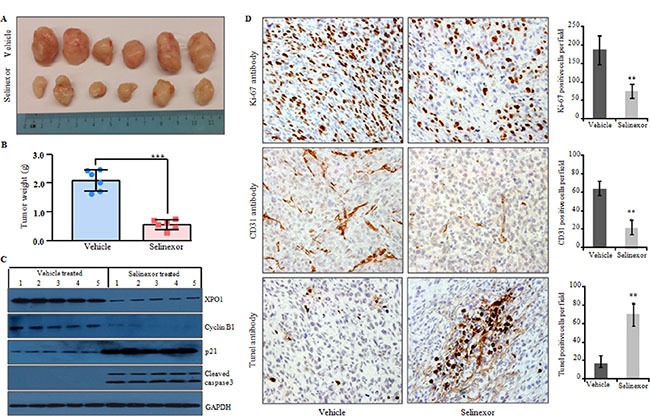
Selinexor significantly reduced tumor growth of LPS141 cells in a xenograft murine model LPS141 cells (2 × 10^6^) were implanted subcutaneously into the right flank of 6-week old male NSG mice. After 14 days, mice were randomly placed into two groups and treated by gavage with either vehicle control or selinexor (10 mg/kg, twice a week X 4 weeks). (**A**) Tumors from mice treated with vehicle versus selinexor (*n* = 6 for each group). Scale in cm. (**B**) Selinexor significantly reduced tumor weight compared to vehicle (dissected tumors). Data are the mean ± SD of the tumors. ***P ≤ 0.0001 (Student's *t-test*). (**C**) Western blot analysis of lysates of LPS141 xenograft tumors from mice treated with selinexor; protein expression of XPO1, cyclin B1, p21, cleaved caspase 3 (GAPDH, internal control). (**D**) Immunohistochemical staining of Ki-67 (proliferation), CD31 (blood vessels) and TUNEL (apoptosis) in liposarcoma xenograft tumors from mice treated with either vehicle or selinexor (original magnification, X 200; objective, X 20. Columns (on the right) show percentage positively stained cells (mean ± SD of three independent experiments). **P ≤ 0.001(Student's *t-test*).

LPS141 liposarcoma cells were injected subcutaneously into the right flank of NSG immunodeficient mice; after 14 days, tumors reached approximately 100 mm^3^ in all the mice. The mice were randomly divide into two groups, and systemic treatment was begin with either selinexor (10 mg/kg dissolved in 100 μl of vehicle orally) or oral vehicle (100 μl) alone, 3 times per week for 4 weeks. Selinexor treatment resulted in significant decreased in tumor volumes and weights compared to the xenograft tumors in mice receiving vehicle alone (Figure 4A and 4B). Western blot analysis of lysates of tumor tissue showed that selinexor decreased the protein levels of XPO1 and cyclin B1 and increased the levels of p21 and cleaved caspase 3 in the mice receiving selinexor compared with tumors in mice treated with vehicle control (Figure 4C). Immunohistochemistry of these tumors showed significantly decreased number of Ki-67 (cell proliferation marker) and CD31 (blood vessels) positive cells and an increase in TUNEL-positive cells (increased apoptosis) in the selinexor treatment group compared to vehicle control group (Figure 4D). Selinexor treatment caused no noticeable morbidity including no significant effect on body weight, numbers of total white blood cells, neutrophils, platelets, haemoglobin, as well as their serum levels of albumin, aspartate aminotransferase, alanine aminotransferase and creatinine in experimental versus control treated mice (Supplementary Table S2).

### Gene expression profiling in selinexor treated liposarcoma cells

To determine global transcriptional consequences of XPO1 inhibition, gene expression profiling of LPS141 liposarcoma cells was performed after 12 hours treatment with either vehicle (DMSO) or selinexor using microarray. A total of 467 genes were down-regulated and 288 were up-regulated (FDR < 0.05) upon selinexor treatment of LPS141 cells as compared to vehicle treatment (Supplementary Figure S2A). Gene set enrichment analysis showed selinexor exposed cells had significant negative enrichment in cell cycle and aurora kinase pathways and positive enrichment in adipogenesis pathway (Supplementary Figures S2B–S2E). The fidelity of the microarray results was confirmed by further validation of 12 randomly selected genes through quantitative RT-PCR. Congruently with the microarray data, mRNA expression levels of cell cycle pathway genes (*CCNB1*, *CCNB2*, *AURKA*, *AURKB*, *CDC25*, *TPX2)* were significantly decreased, whereas mRNA levels of adipogenesis pathway genes (*CEBPA*, *LPL*, *PPARG2*, *RB1, KLF6*, *IGFBP5*, *FBXW7*, *CEBPD, DDIT3)* were significantly increased in LPS141 cells treated with selinexor compared to vehicle (Supplementary Figure S2F).

### Inhibition of XPO1 induced cytotoxicity in liposarcoma cells by inducing insulin-like growth factor binding protein 5 (IGFBP5)

**Figure 5:**
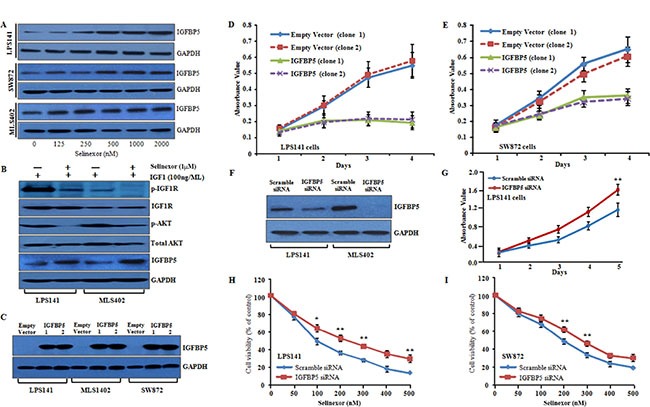
Inhibition of XPO1 induced cytotoxicity by re-expressing IGFBP5; and IGFBP5 overexpression reduces cellular proliferation, migration and invasion in liposarcoma cell lines (**A**) IGFBP5 protein expression by western blot analysis in LPS141, SW872 and MLS402 following selinexor treatment (0–2000 nM, 24 h) (GAPDH, internal control). (**B**) LPS141 and MLS402 cells were serum-starved overnight, treated with selinexor (1000 nM) for 2 h and then stimulated with human IGF-1 (100 ng/ml) for 10 minutes, and the proteins were analyzed by western blot using indicated antibodies. (**C**) Western blot shows overexpression of IGFBP5 protein in LPS141, MLS402 and SW872 cells stably transfected with *IGFBP5*expression vector compared to empty vector (GAPDH, loading control). Clone 1 and 2 are two separate clones that stably express IGFBP5. (**D** and **E**) Overexpression of IGFBP5 in LPS141 and SW872 cells exhibited decreased cell growth in liquid culture. Clones 1 and 2 were two different stable clones expressing IGFBP5. For control, two separate clones containing empty vector were used. Data represent mean ± SD; *n* = 4. **P ≤ 0.001; ***P ≤ 0.0001 (Student *t* test). (**F**) Western blot analysis verified silencing of IGFBP5. (**G**) MTT assay showed that knockdown of IGFBP5 increased cell proliferation in liquid culture. (**H** and **I**) LPS141 and SW872 cells were transfected either with *IGFBP5* siRNA or scramble siRNA. These cells were exposed to different concentration of selinexor for 48h, and growth inhibition was measured by MTT assay. Data represent mean ± SD; *n* = 4. **P ≤ 0.001. Data for G, H and I represent mean ± SD of three independent experiments done in triplicates. **P < 0.001 (Student's *t-test*).

Insulin-like growth factor 1 receptor (*IGF1R)* is expressed in a wide range of tumors including liposarcoma; and *IGF1R* signaling is crucial for tumor formation and survival of malignant cells [23]. A recent study showed that the combination of *IGF1R* and *CDK4* inhibitors synergistically reduced the cell proliferation of liposarcoma cells [24]. We found in our microarray data that two *IGF-1* binding proteins (*IGFBP5* and *IGFBP6*) were up-regulated in selinexor treated LPS141 cells (Supplementary Figure S2A). Many previous reports showed that *IGFBP5* can act either as a tumor suppressor or oncogene in a tissue-specific context but nothing is known about the role of *IGFBP5* in human liposarcoma [25–27]. RNA-sequencing analysis confirmed down-regulation of *IGFBP5* mRNA in liposarcoma cell lines compared to human adipose tissue (Supplementary Figure S3A, manuscript in preparation). Further, microarray data analysis (GSE21122) also showed down-regulation of *IGFBP5* compared to human normal fat samples (Supplementary Figure S3B). The up-regulation of *IGFBP5* mRNA and protein upon treatment with selinexor was further verified both at the mRNA and protein levels in liposarcoma cell lines (Figures 5A and Supplementary Figure S3C). Interestingly, treatment of LPS141 and MLS402 cell lines with selinexor resulted in attenuation of phosphorylation of both IGF-1R and AKT after stimulation with IGF-1 (Figure 5B).

We generated stable clones of LPS141, SW872 and MLS402 cells expressing either an empty vector or *IGFBP5* expression vector (Figure 5C). Overexpression of *IGFBP5* in LPS141 and SW872 cells significantly (*P* < 0.001) inhibited cell growth in liquid culture (Figure 5D and 5E), as well as clonogenic growth in soft agar (Supplementary Figure S3D and S3E) compared with empty vector. Silencing of *IGFBP5* resulted in significant increase in the growth of LPS141 cells in liquid culture (Figure 5F and 5G). Furthermore, knockdown of *IGFBP5* in LPS141 and SW872 cells partially rescued the inhibitory effect of selinexor treatment (Figure 5H and 5I).

### Role of aurora kinases in liposarcoma

**Figure 6:**
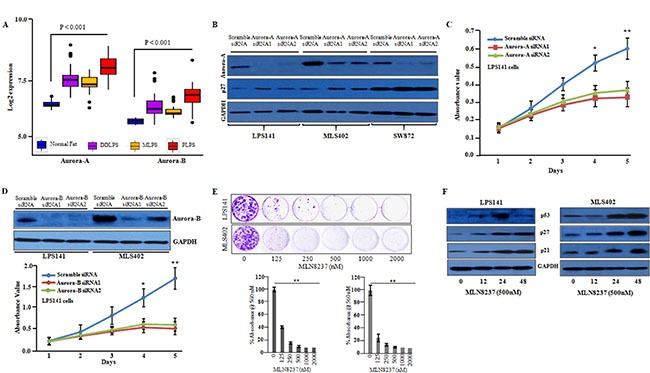
Inhibition of aurora-A and aurora-B decreased the cellular growth of liposarcoma cells (**A**) Microarray data (GSE21122) showed that *aurora-A* and *aurora-B* were significantly upregulated in liposarcoma patient samples compared to normal fat. (**B**) Western blot analysis confirmed knockdown of *aurora-*A in siRNA1 and siRNA2 transfected cells compared to scramble siRNA in LPS141, MLS402 and SW872 cells. (**C**) *Aurora-A* siRNAs suppressed the growth of LPS141 cells in liquid culture. Data represent mean ± SD; *n* = 4. *P ≤ 0.01; ***P ≤ 0.001 (Student *t* test). (**D**) Western blot analysis confirmed silencing of *aurora-B* in LPS141 and MLS402 cells and a*urora-B* knockdown suppressed the growth of LPS141 cells in liquid culture. (**E**) Soft agar assay: MLN8237 significantly inhibited clonogenic growth of LPS141 and MLS402. Data represent mean ± SD of three independent experiments done in triplicates. **P < 0.001 (Student's *t-test*). (**F**) LPS141 and MLS402 cells were treated with MLN8237 (500 nM) for 12, 24, 48 h; and the protein levels of p53, p21, p27 were analyzed by western blot. GAPDH was used to ensure equal loading of lysate.

Our microarray data showed that aurora kinase pathway was one of the most significantly enriched pathways in the down-regulated genes in liposarcoma cells upon selinexor treatment compared to vehicle exposure (Supplementary Figures S2A and S2C). Recently, several aurora kinase inhibitors have entered Phase I and II clinical trials against different types of cancers [28–30]. Analysis of our RNA-sequencing data displayed robust expression of both *aurora-A* and *aurora-B* mRNA in liposarcoma cell lines compared to normal human adipose tissue (Supplementary Figures 4A and 4B, manuscript in preparation). In silico analysis also confirmed that *aurora-A* and *aurora-B* mRNAs were significantly upregulated in human liposarcoma microarray data (GSE21122) (Figure 6A). Expression of *aurora-A* and *aurora-B* was silenced in liposarcoma cells using siRNA which resulted in a significant decreased in cell growth of LPS141 and MLS402 in liquid culture (Figure 6B–6D, Supplementary Figure S4C). Selective inhibitors of *aurora-A* (MLN8237) and *aurora-B* (ZM447439) inhibited auto-phosphorylation of *aurora-A* and *aurora-B* and decreased growth in liquid (Supplementary Figure S4D and S4E) as well as clonogenic growth (Figure 6E) of LPS141 and MLS402 cells. Also, these drugs lowered the level of cyclin B1 (Supplementary Figure S4F) and markedly increased the protein expression of p53, p27 and p21 in LPS141 and MLS402 cells (Figure 6F).

## DISCUSSION

Liposarcoma is one of the most common soft tissue sarcoma causing substantial morbidity and mortality [3, 31, 32]. Inhibition of nucleo-cytoplasmic transport by natural and synthetic products is a therapeutic approach [33]. Recently, Selective Inhibitors of Nuclear Export compounds have been developed [17, 22] and showing anti-tumor activity against various human malignancies *in vitro* and in murine human cancer xenograft models with minimal toxicities [15–17, 34].

Present study shows prominent expression of *XPO1* protein in different histological subtypes of liposarcoma in patient samples and cell lines compared to benign lipomas. Immunofluorescence experiment data confirmed that *XPO1* is localized at the nuclear pore in liposarcoma cells. Also, microarray database GSE21122 indicated significant increased *XPO1* mRNA expression in different subtypes of liposarcoma samples compared to normal fat. These data suggest that robust expression of *XPO1* may have a potential role in the pathogenesis or progression of liposarcoma. Silencing of *XPO1* suppressed the growth of several liposarcoma cell lines. Further, selinexor caused growth inhibition, cell-cycle arrest with upregulation of p21, p27, p53 and downregulation of cyclin B1, cyclin E and survivin. The drug increased apoptosis as shown by upregulation of cleaved caspase 3, cleaved PARP and BAX, as well as annexin V positive staining of liposarcoma cells. Well-differentiated and dedifferentiated liposarcomas have 12q13-15 amplification resulting in MDM2 overexpression. MDM2 can inactivate p53. We found that selinexor increased the expression p53 and its target genes (e.g., p21 and BAX) proteins in LPS141 liposarcoma cells (*MDM2* amplification) without altering the expression of MDM2 (Figure 3B and Supplementary Figure S5A). This observation is consistent with a previously published study [35]. Of note, selinexor exposure (≤ 2000 nM) did not affect *p53*, *MDM2*, *CDKN1A* mRNA levels suggesting that the drug post-transcriptionally increased the level of these proteins (Supplementary Figure S5B). Of interest, a recent study showed that selinexor induced cell cycle arrest and apoptosis of liposarcoma cells irrespective of their *p53* expression or mutational status [35].

Importantly, selinexor (10 mg/kg orally, three times per week at a non-toxic dose) significantly inhibited growth of dedifferentiated liposarcoma (LPS141) xenografts in NSG mice associated with reduced cellular proliferation (Ki-67 staining) and an increase in apoptosis (Tunel staining) in the tumors. Recently, a phase 1 clinical trial of selinexor in patients with advanced solid tumors including liposarcoma [36, 37] showed a favorable anti-tumor effect of selinexor in 78% (14 of 18) of the liposarcoma patients, with a well-tolerated dose of 35 mg/m^2^ (approximately 60 mg flat dose) [37]. Taken together, these results strongly indicate that inhibition of *XPO1* might be a valuable treatment approach for this disease.

Our microarray data analysis showed that *IGFBP5* expression was markedly increased in liposarcoma cells after treatment with selinexor. *IGFBP5* is the most conserved member of the *IGFBPs* family and is frequently deregulated in human malignancies such as neuroblastoma [25], osteosarcoma [27, 38], breast [26, 39, 40] as well as head and neck squamous cell carcinoma [41]. *IGFBP5* modulates cellular functions either in an IGF-dependent manner through IGF1R signaling or in an IGF-independent manner [42]. Notably, we found that selinexor increased expression of *IGFBP5* both at the mRNA and protein levels in liposarcoma cells. We provide evidence that *IGFBP5* can acts as a tumor suppressor in liposarcoma cells in an *IGF-1* dependent manner; and selinexor can markedly up-regulate the expression of this tumor suppressor protein. In addition, microarray data showed significant enrichment of the aurora kinase pathway in liposarcoma cells. Overexpression and gene amplification of *aurora-A* and *aurora-B* in liposarcoma have been reported to correlate with tumor grade and prognostic markers [43–46]. We showed that selinexor can significantly decrease levels of *aurora-A* and *aurora-B* mRNA. Also, inhibition of aurora kinases either through siRNA or selective inhibitors of aurora-A and aurora-B suppressed the cell growth of liposarcoma cells in both liquid culture and soft agar associated with decreased cyclin B1 and increased expression of tumor suppressor proteins, p53, p27, and p21.

Taken together, our data demonstrated that in preclinical studies, selinexor is a potent therapeutic agent against liposarcoma. This activity is probably mediated by a number of pathways as suggested by our studies. Clinical trials of this novel agent in liposarcoma are clearly warranted.

## MATERIALS AND METHODS

### Cell culture and reagent

Human liposarcoma cell line SW872 (undifferentiated liposarcoma) was purchased from American Tissue Type Culture Collection (ATCC, Rockville, MD, USA). SA-4 was generously provided by Dr. Ola Myklebost; LPS141 and LP6 were kindly provided by Dr. Jonathan A. Fletcher at Brigham and Women's Hospital (Boston, MA, USA); FU-DDLS-1 was a gift from Dr. Nishio. GOT3 and MLS402 were generous gifts from Dr. Aman. T7778 and T1000 were kindly provided by Dr. Pedeutour. All cell lines were cultured and maintained in RPMI1640 containing 10% fetal bovine serum (FBS) and 1% penicillin-streptomycin (Invitrogen, Carlsbad, CA) at 37°C in a humidified atmosphere with 5% CO_2_. Human IGF-1 was from PROSPEC (East Brunswick, NJ). Selinexor (KPT-330) and Pluronic F-68 were kindly provided by Karyopharm Therapeutics (Newton, MA, USA). For *in vitro* studies, selinexor was dissolved in dimethyl sulfoxide (Sigma-Aldrich) to a concentration of 20 mM/L. For *in vivo* study, selinexor was dissolved in vehicle solution [0.6% (w/v) aqueous Pluronic F-68 and 0.6% (w/v) PVP-K29/32 (Karyopharm Therapeutics) in nuclease-free water.

### Western blot analysis

Cell lysates were prepared using Protein Extraction reagent (Thermo Scientific) containing protease inhibitor cocktail (Roche Molecular Biochemical). Western blots were performed as described previously [47]. Briefly, proteins were transferred to a polyvinylidene fluoride membrane (Immobilion, Millipore), blocked with 5% non-fat milk and incubated with indicated antibodies overnight in the cold room. Blots were washed and incubated with HRP-conjugated secondary antibodies for 1 hr. Super-Signal West Pico and West Dura Chemiluminescent substrate (Pierce Biotechnology, Rockford, IL, USA) were used for protein detection. Antibodies against XPO1/CRM1 (H300), C-Myc, p27 (C-19), Bax (N20), Cyclin D1 (A-12) were purchased from Santa Cruz Biotechnologies (Dallas, TX, USA). Antibodies against cyclin B1, p21, cleaved PARP, cleaved caspase-9, cleaved caspase-3, p-IGF1R, IGF1R, GAPDH and α-tubulin were obtained from Cell Signaling Technology (Danvers, MA, USA). Antibody against β-actin and IGFBP5 were from Sigma-Aldrich (St. Louis, MO).

### RT-PCR analysis and quantitative real-time PCR (qRT-PCR)

Total RNA was isolated from liposarcoma cell lines (LPS141, SW872, MLS402 and SA4) with the RNeasy mini kit (Qiagen GmbH, Hilden, Germany) according to manufacturer's protocol. 1 ug of total RNA was used for complementary DNA (cDNA) synthesis using one step cDNA synthesis kit (Life Technologies Inc., Gaithersburg, MD, USA). For quantitative real-time PCR amplification, KAPA SYBR Green was used. Real-time PCR was performed using KAPA SYBR master mix (KapaBiosystems, Woburn, MA, USA) on an ABI 7900 Fast real-time PCR system (Applied Biosystems, USA) following the supplier's protocol. Thermal conditions for real-time qRT-PCR were set at 95°C for 10 min for initial denaturation, followed by 40 cycles of PCR with denaturation at 95°C for 15 seconds and annealing/extension at 60°C for 1 min. Primers used for qRT-PCR for selected genes are shown in Supplementary Table S1.

### Indirect immunofluorescence assay

For immunofluorescence experiments, LPS141, SW872, MLS402 AND SA4 cells were fixed with ice-cold methanol and incubated with the murine monoclonal XPO1/CRM1 antibody (1:500 dilution) at room temperature for 2 hr. Cells were washed and incubated with secondary antibody [anti-murine IgG conjugated with Alexa Fluor 594 (Life Technologies, USA)]. 4′, 6-diamidino-2-phenylindole (DAPI; Sigma-Aldrich, St. Louis, MO) was used to stain the nucleus of the cells. Slides were finally washed and mounted in mounting medium (Sigma-Aldrich, St. Louis, MO) and images were captured using Nikon Eclipse E 400 microscope (Nikon, Fukok, Japan).

### Cell proliferation assay (MTT assay)

Anti-proliferative effect of selinexor against liposarcoma cells was determined using colorimetric assays as described [48]. Briefly, 4 × 10^3^ liposarcoma cells were seeded in quadruplets in 96-well plates either in the presence or absence of selinexor at 37°C in a CO_2_ incubator. At the conclusion of the experiment, 20 μl of 3-(4,5-dimethylthiazol-2-yl)-2,5-diphenyltetrazolium bromide (MTT; Sigma-Aldrich) was added to each well and incubated at 37°C in a CO_2_ incubator for 2hr and then dissolved in 100 μl of stop solution (SDS-HCl). Absorbance was measured at 570 nm using a microplate reader (Infinite 200; Tecan, San Jose, CA, USA). Dose-response curves were plotted to calculate half-maximal inhibitory concentrations (IC50) for selinexor by GraphPad Prism4 (Graph Pad Software, San Diego CA, USA).

### Colony formation assay

Liposarcoma cells (1 × 10^3^) were seeded into 6-well plates in triplicates in complete medium. After two days, media were supplemented with varying concentrations of selinexor. After 2 weeks, colonies were fixed with 5% glutaraldehyde and stained with crystal violet. For quantitative measurement, colonies were dissolved in 200 ul of DMSO and absorbance was measured at 570 nm using a microplate reader (Infinite 200; Tecan, San Jose, CA, USA).

### Cell cycle analysis

To determine the effect of selinexor on cell cycle, liposarcoma cells were cultured with either diluent control (DMSO) or various concentrations of selinexor at indicated time points as described earlier [47]. Briefly, cells were trypsinized, washed with ice-cold PBS and fixed with 70% chilled ethanol. Cells were stained with PI solution (50 μg/ml PI, Triton X-100 (1%), 20 ug/ml DNase-free RNase A in PBS) for 30 min at 37°C in dark and analyzed by LSR-II flow cytometer (Becton-Dickinson, San Jose, CA, USA).

### Annexin V and propidium iodide (Annexin V-PI) apoptosis analysis

Annexin V-PI staining was performed using flow cytometric analysis as previously described. Briefly, 1 × 10^6^ cells were cultured with either diluents control (DMSO) or varying concentrations of selinexor in 6 well plates for 72 h and staining was performed using Apoptosis Detection Kit II (BD Biosciences, USA). Cells were washed twice with 1X ice-cold phosphate-buffered saline (PBS; Life technologies, USA), trypsinized and centrifuged. Cells were again washed with 1X PBS, resuspended in 1X binding buffer containing 5 ul of FITCI conjugated Annexin V and 5 ul of PI for 20 min in dark. The samples were analyzed using LSR-II flow cytometer (BD, San Jose, CA, USA).

### Liposarcoma xenograft murine model

Six-week-old male NSG mice were injected with LPS141 (2 × 10^6^) cells suspensions mixed with matrigel (BD Biosciences) (1:1) in 200 μl of total volume and injected subcutaneously on the flank. Treatment was initiated 2 weeks after cell implantation. The mice were randomly divided into two groups (6 mice per group) to receive either vehicle control (0.6% w/v aqueous Pluronic F-68) or selinexor (10 mg/kg in 100 μl of the vehicle, 3 times per week). Tumor diameters were measured with a caliper, and tumor volumes were calculated by the formula (V = π/6 × Dl × Ds^2^), where V is volume, Dl is the largest diameter, and Ds is the smallest diameter [49]. Mice were fed with Nutri-Cal (Tomlyn) thrice a week during treatment to provide good nutrition. All mice were sacrificed at the end of the study. Tumors were dissected and weighed. One-half of each tumor was fixed in 10% formaldehyde for immunohistochemistry and the other half was immediately kept in −80°C for subsequent RNA and protein analysis. We also analyzed peripheral blood for blood counts and serum chemistry after the 28-days of treatment (Supplementary Table S2). The animal studies were approved by the National University of Singapore (NUS) Institutional Animal Care and Use Committee (IACUC).

### Immunohistochemistry

Tissue microarray slides were purchased from Super Bio Chips. Tumor xenografts were fixed in 10% formaldehyde, embedded in paraffin and cut into 4 μm thick sections. Immunohistochemistry was performed as described [47, 49]. Briefly, endogenous peroxidase activity was blocked using 3% hydrogen peroxide for 10 min and antigen retrieval was done using retrieval buffer (pH 6). Slides were blocked with 10% goat serum. Antibodies against XPO1, CD31, Ki-67 and Tunel were used. Immunostaining was assessed by counting > 500 cells from 5 random fields of each specimen under ×400 magnification in the best-stained tumor area of each section as described previously [3, 47].

### Microarray analysis

Total RNA was extracted LPS141 cells after 12 hours treatment with either vehicle (DMSO) or selinexor using RNeasy mini kit (Qiagen, Valencia,CA) according to manufacturer's protocol for microarray experiments as described previously [47]. RNA Nano chip on Agilent Bioanalyzer 2100 (Agilent Technologies, Inc., Santa Clara, CA) was used to check integrity and quantity of RNA samples. cDNA and cRNA was synthesized using Illumina Total Prep RNA Amplification Kit (Illumina). Human gene HT-12 v4 Expression beads chip kits (47,000 probe spotted genes) from Illumina (Santa Clara, CA) were used.

### RNA interference

To obtained knockdown of *XPO1* in liposarcoma cell lines for *in vitro* studies, human *XPO1* gene specific shRNAs and a non-targeting shRNA were cloned into a lentiviral vector. For stable knockdown, lentiviral particles were generated according to manufacturer's protocol. Cells were infected with lentivirus particles at a MOI of 20 with 8 μg/ml (Sigma-Aldrich) for 24 h, and stable cells were selected using puromycin for 1–2 weeks.

Human *IGFBP5*, *aurora-A* and *aurora-B* gene specific siRNA oligos (siGENOME) including scramble oligos were purchased from Dharmacon (Coralville, USA). LPS141, MLS402 and SW872 cells were transfected with 30 pmol siRNA using Lipofectamine RNAiMax (Life Technologies, USA) according to the manufacturer's protocol. After 48 hours of transfection, cells were used for qRT-PCR; western blotting confirmed knockdown. Each experiment was performed at least in triplicate on three different occasions.

### Statistical analysis

For *in vitro* and *in vivo* experiments, we evaluated the statistical significance of the difference between two groups using two-tailed Student *t-test*. Asterisks in the figures represent significant differences between experimental groups in comparison to controls ((*p < 0.01, **p < 0.001, ***p < 0.0001). Data points in figures represent the means ± SD (standard deviation).

## SUPPLEMENTARY MATERIALS FIGURES AND TABLES


