# Supplementary material for: Molecular mechanism and therapeutic implications of selinexor (KPT-330) in liposarcoma
Source: Oncotarget. 2016 Nov 21;8(5):7521–32. doi: 10.18632/oncotarget.13485 (PMC5352339; doi:10.18632/oncotarget.13485)
Supplement: Supplementary file 1 [file oncotarget-08-7521-s001.pdf]

Molecular mechanism and therapeutic implications of selinexor (KPT-330) in liposarcoma

Supplementary Materials

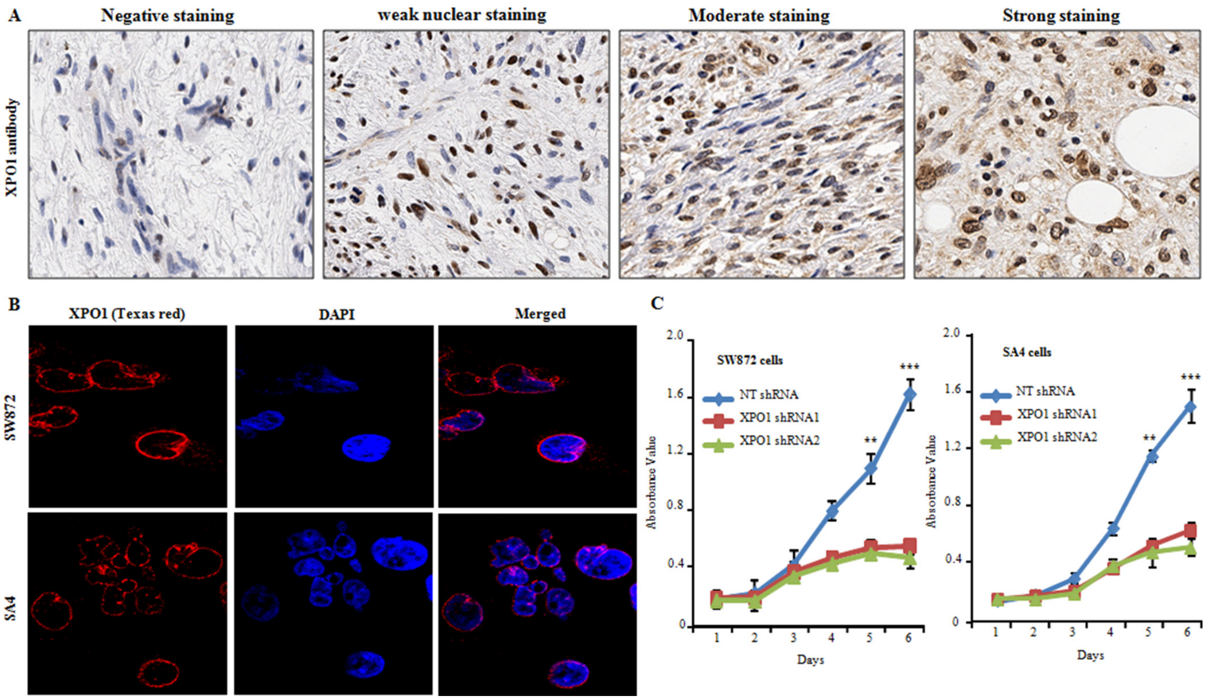

Supplementary Figure S1: Expression of *XPO1* in human liposarcoma samples and cell lines; and silencing of *XPO1* decreased cellular growth of liposarcoma cells in liquid culture. (**A**) Representative photomicrographs showed negative, low, moderate and strong nuclear staining of XPO1 protein in liposarcoma samples (original magnification, X200; objective, X20). (**B**) Immunofluorescence showed nuclear localization of endogenous XPO1 protein expression in fixed/permeabilized SW872 and SA4 liposarcoma cells; DAPI stained nuclei. (**C**) *XPO1* shRNA inhibited cell proliferation in liquid culture (SW872 and SA4). Data represent mean ± SD; $n = 4$. **$P \leq 0.001$, ***$P \leq 0.0001$.

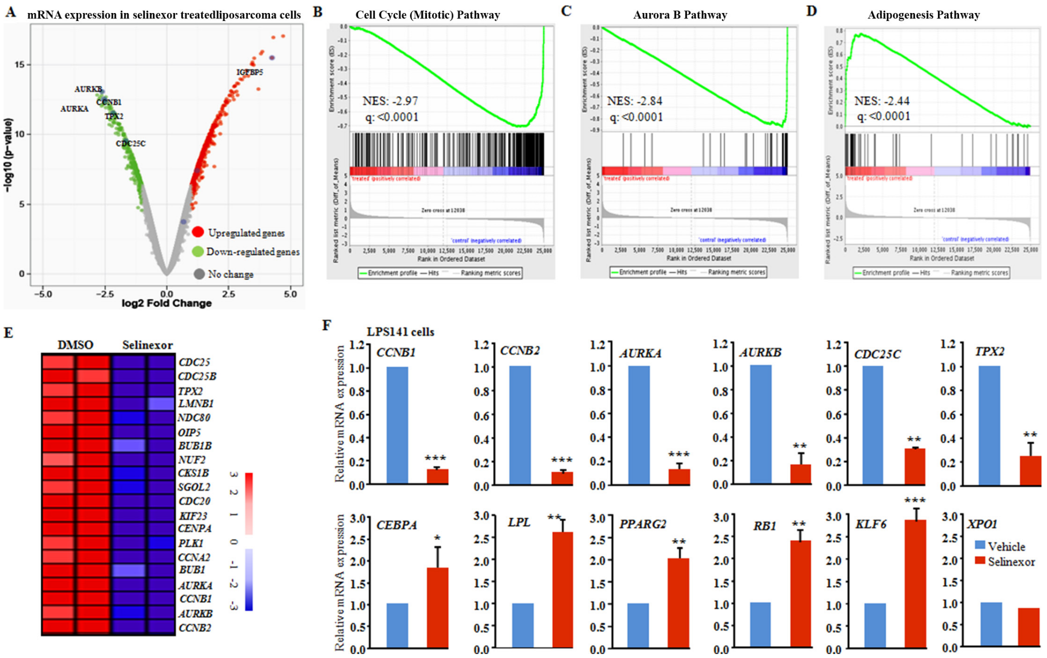

Supplementary Figure S2: Genome-wide transcriptome analysis of selinexor treated liposarcoma cells. (**A**) Microarray data: Volcano plot shows differentially expressed genes for LPS141 cells treated with either vehicle (DMSO) or 1000 nM selinexor for 12 h. Red and green dots represent up-regulated and down-regulated genes, respectively in the selinexor treated cells compared to control. Grey dots represent no change. (**B**–**D**) Gene set enrichment analysis showed negative enrichment of cell cycle, aurora kinase B pathway, and positive enrichment of adipogenesis. NES indicates normalized enrichment score; and *q* = false discovery rate. (**E**) Microarray data: Heat-map of the top 20 significantly down-regulated genes in the treatment group. (**F**) qRT-PCR validation of 12 selected genes identified as differentially expressed by microarray. Expression of each gene was normalized to *GAPDH* as a reference. Figures are representative of 3 replicates. Data represent mean ± SD, $n = 3$. $P \leq 0.01$, **$P \leq 0.001$, ***$P \leq 0.0001$.

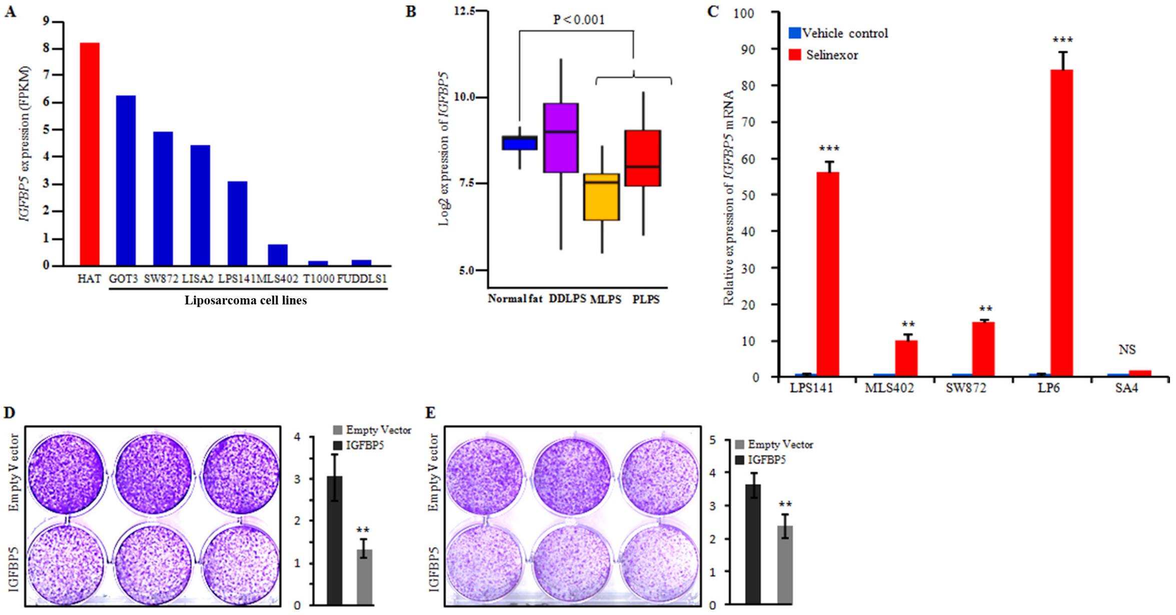

Supplementary Figure S3: Inhibition of *XPO1* induced cytotoxicity by re-expressing insulin growth factor binding protein 5 *(IGFBP5)*. (**A**) RNA-sequencing analysis of IGFBP5 expression in normal human adipose tissue (HAT) and liposarcoma cell lines. (**B**) Microarray data (GSE21122) for 46 DDLPS, 23 PLPL, 20 MLPS samples and 9 normal fat samples. *IGFBP5* mRNA was significantly ($P < 0.001$) down-regulated in human MLPS and PLPS samples compared to normal human fat samples. (**C**) Liposarcoma cells were cultured with either vehicle control or selinexor (1000 nM, 24h); qRT-PCR of the cDNAs was performed using *IGFBP5* primers. *GAPDH* primers were used for normalization. (**D**–**E**) Overexpression of *IGFBP5* resulted in decreased clonogenic growth of LPS141 and SW872 cells compared to empty vector control cells. Colonies were stained with crystal violet. Representative photomicrograph and quantitative analysis showed a reduction in clonogenic growth. Data for (C–E) represent mean ± SD of three independent experiments done in triplicates. **$P \leq 0.001$; ***$P \leq 0.0001$ (Student *t* test).

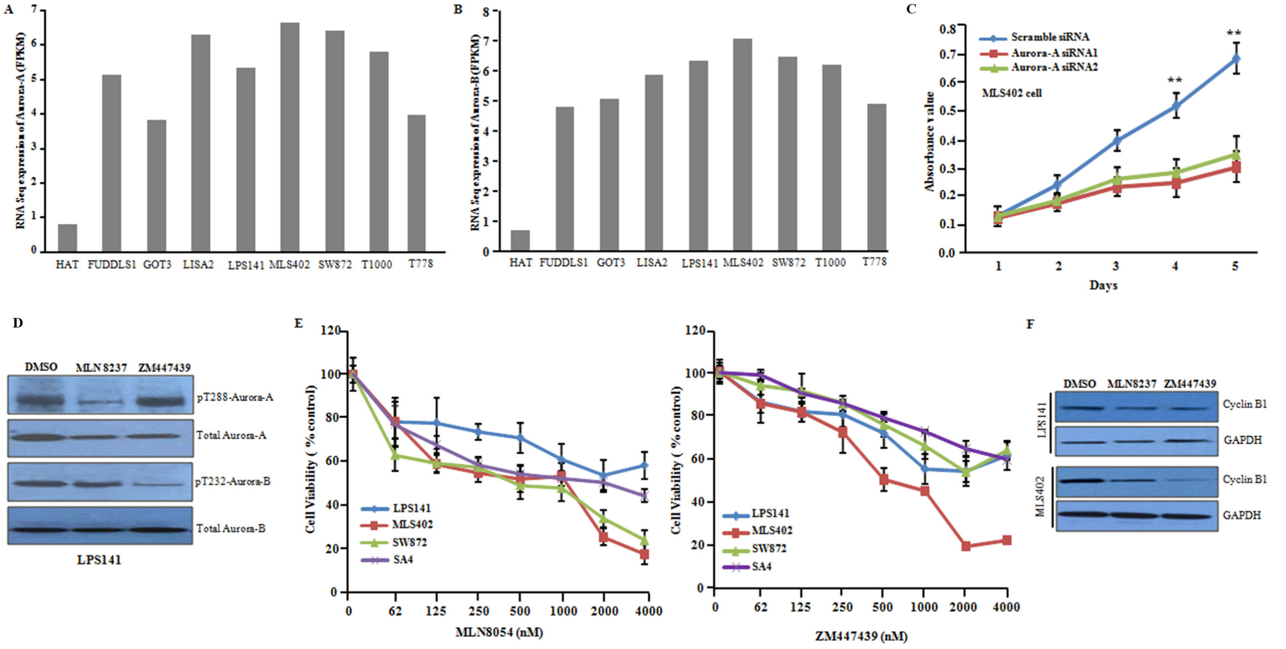

Supplementary Figure S4: Silencing aurora-A and aurora-B inhibited cell growth in liquid culture. (**A**–**B**) RNA-sequencing data showed upregulation of *aurora-A* and *aurora-B* mRNA in liposarcoma cell lines compared with normal human adipose tissue (HAT). (**C**) Knockdown of *aurora-A* in MLS402 cells slowed their growth. Data represent mean ± SD of three independent experiments done in triplicates. $^{**}P < 0.001$ (Student's *t*-test). (**D**) Western blots analysis showing the selectivity of MLN8054 and ZM447439 for inhibition of p-aurora-A and p-aurora-B, respectively. (**E**) Liposarcoma cells were treated with vehicle control or increasing doses of inhibitors of either *aurora-A* (MLN8237; 0-4000 nM for 72 h) (left panel) or *aurora-B* (ZM447439; 0–4000 nM for 72 h) (right panel). Inhibition of cellular growth was determined by MTT assay. Data represent mean ± SD of three independent experiments done in triplicates. (**F**) Liposarcoma cells were treated with either MLN8054 or ZM447439 (500 nM, 24 h); and the protein levels of cyclin B1 and GAPDH were determined on Western blots.

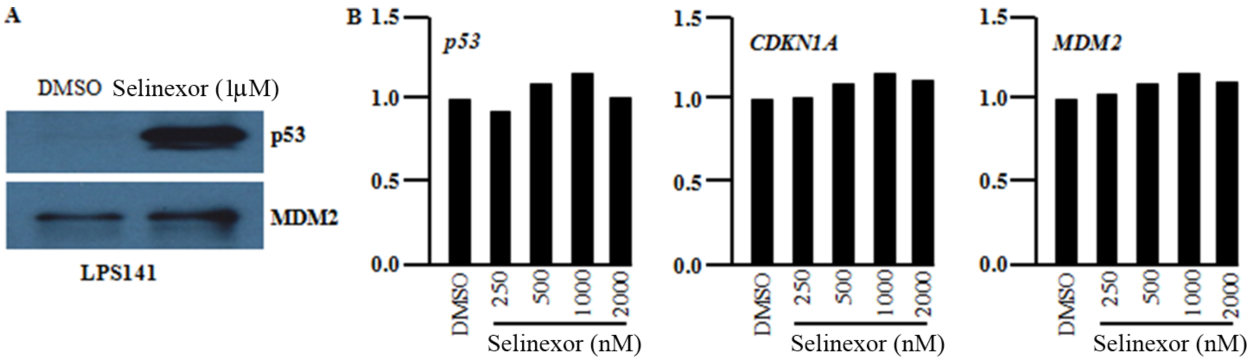

Supplementary Figure S5: Selinexor induced p53 without altering *MDM2*. (**A**) LPS 141 cells were cultured in presence of selinexor (1000 nM) for 24 h. Cell lysates were prepared and subjected to western blot analysis using p53 and MDM2. (**B**) LPS141 cells were exposed to different concentration of selinexor for 24 h and total RNA was extracted and converted into cDNA. The qRT-PCR analysis was performed for mRNA expression of p53, *CDKN1A* and *MDM2*.

Supplementary Table S1: Selected gene primers for real-time quantitative PCR

Gene name	Forward primer	Reverse primer
XPO1	5′-CTCGTCAGCTGCTTGATTTC-3′	5′-CTCTTGTCCAAGCATCAGGA-3′
CCNB1	5′- CATGGTGCACTTTCCTCCTT-3′	5′- CAGGTGCTGCATAACTGGAA-3′
CCNB1	5′-CCTCCCTTTTCAGTCCGC-3′	5′-CTCCTGTGTCAATATTCTCCAAATC-3′
CDC25C	5′-CTTCCTTTACCGTCTGTCCAG-3′	5′-CCAAGTTTCCATTGTCATCCC-3′
AURKA	5′-ATCTGTGGTGCATTGGAGTG-3′	5′-CATACAAACACACGCACCCG-3′
AURKB	5′-CAGAGAGATCGAAATCCAGGC-3′	5′-CCTTGAGCCCTAAGAGCAGAT
TPX2	5′-TCACTCGTGGTGTGGACTTC-3′	5′-AGCATCGCAGAATCCATTGG-3′
IGFBP5	5′-AGCACAGATACCCAGAACTTCTCC-3′	5′-TCCATTTCTCTACGGCAGGG-3′
IGFBP5	5′-TGACCGCAAAGGATTCTACAAG-3′	5′- CGTCAACGTACTCCATGCCT-3′
KLF6	5′-CGGACGCACACAGGAGAAAA-3′	5′-CGGTGTGCTTTCGGAAGTG-3′
KLF6	5′-CTGCCGTCTCTGGAGGAGT-3′	5′-TCCACAGATCTTCCTGGCTGTC-3′
GAPDH	5′-TGAAGGTCGGAGTCAACGGAT-3′	5′-GTCATGAGTCCTTCCACGATA-3′

Supplementary Table S2: Analysis of murine blood samples after treatment with vehicle and selinexor

Blood and serum analysis	Reference range	Vehicle	Selinexor (10 mg/kg)
WBC (× 1000 per µl)	1–12.2	5.1 ± 0.38	4.3 ± 0.76
Neut (× 1000 per µl)	0–2.5	2.6 ± 0.31	2.0 ± 0.32
HCT (%)	34–53	37.3 ± 3.0	40.6 ± 2.3
PLT(× 1000 per µl)	625–2241	1950 ± 322	1621 ± 513
ALB (g/L)	25–30	23.4 ± 3.2	27.6 ± 3.3
AST (U/L	54–298	97.4 ± 38	131 ± 30
ALT (U/L	17–77	28 ± 14	46.3 ± 24
CREAT (mg/dl)	0.2–0.9	0.18 ± 0.4	0.25 ± 0.12

Mice were randomly divided into two groups (six mice per group) and orally treated either with vehicle (0.6% w/v aqueous Pluronic F-68) or Selinexor (KPT-330; 10mg/kg, thrice weekly for 4 weeks). Mice were given Nutri-Cal (tomlyn) during therapy to allow robust nutrition. Data represent mean ± SD of NOD/SCID mice.
Abbreviations: WBC = white blood cell; Neut = neutrophil; HCT = haematocrit; PLT = platelet; ALB = albumin; AST = aspartate aminotransferase; ALT = alanine aminotransferase; CREAT = creatinine.
